# Occult Thyroid Carcinoma without Malignant Thyroid Gland Findings during Preoperative Examination: Report of Three Cases

**DOI:** 10.1155/2020/4249067

**Published:** 2020-04-10

**Authors:** Soji Toda, Hiroyuki Iwasaki, Nobuyasu Suganuma, Yoichiro Okubo, Hiroyuki Hayashi, Katsuhiko Masudo, Hirotaka Nakayama, Munetaka Masuda

**Affiliations:** ^1^Department of Breast and Endocrine Surgery, Kanagawa Cancer Center, Yokohama, Japan; ^2^Department of Pathology, Kanagawa Cancer Center, Yokohama, Japan; ^3^Department of Breast and Thyroid Surgery, Yokohama City University Medical Center, Yokohama, Japan; ^4^Department of Surgery, Yokohama City University School of Medicine, Yokohama, Japan

## Abstract

Occult thyroid carcinoma preceded by clinical manifestations and findings from extrathyroidal tumors is rare. The lack of malignant findings in the thyroid during the preoperative examination makes diagnosis difficult. We encountered a 71-year-old man with a primary ectopic thyroid carcinoma causing superior vena cava syndrome. Although no malignant findings were found in the thyroid gland, biopsy of bone metastases led to the diagnosis of thyroid cancer. HE staining of bone metastases revealed nuclear features of papillary carcinoma, and immunostaining was positive for thyroglobulin and PAX-8. The second case involved an 84-year-old man with a mediastinal tumor and suspected thyroid cancer because of high thyroglobulin levels in blood. The pathological tumor finding was papillary thyroid cancer. The last case was that of a 56-year-old woman lacking preoperative thyroid examination malignant findings, but with cervical lymph node metastasis. The thyroglobulin level of the lymph node puncture fluid was useful for preoperative diagnosis. We performed total thyroidectomy plus bilateral modified neck dissection. Pathology revealed a 1 mm papillary carcinoma in the left lobe. All of these cases were difficult to diagnose. However, we combined the results of various tests such as radiographic imaging, blood tests, and immunohistological tests to diagnose our patients.

## 1. Introduction

Papillary thyroid cancer is the most common thyroid cancer and is likely to metastasize to the cervical lymph nodes often found in a thyroid mass. However, some thyroid cancers do not show malignant findings in the thyroid during preoperative examinations. These occult carcinomas are rare, and their diagnosis is difficult. In this paper, we report the cases of three patients with occult thyroid cancer preceded by clinical manifestations and findings from extrathyroidal tumors.

## 2. Case Presentation

**Figure 1:**
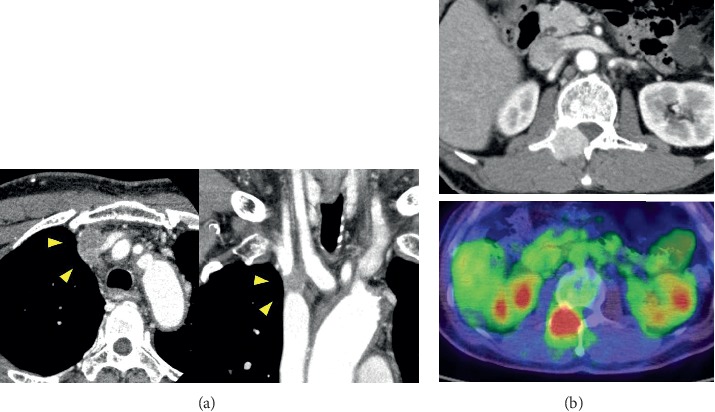
CT and PET-CT findings. (a) Computed tomography showing a 27 mm lump on the right side of the upper mediastinum that is invading the superior vena cava. (b) PET-CT showing metastasis of the lumbar vertebrae.

Case 1 is a 71-year-old man who had facial edema for 9 months. He had jugular venous distention and eyelid edema. An enhanced computed tomography (CT) scan showed a 27 mm lump on the right side of the upper mediastinum, stenosis of the superior vena cava, and dilation of surrounding collateral vessels (Figure 1(a)). We diagnosed the patient as presenting superior vena cava syndrome due to superior mediastinal tumor.

**Figure 2:**
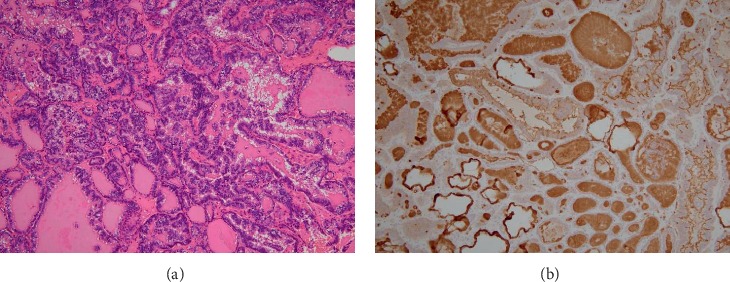
Bone biopsy results of the lumbar vertebrae. HE staining showing a papillary structure and positive immunohistochemistry for thyroglobulin. (a) HE staining ×100. (b) Immunohistochemistry for thyroglobulin ×100.

Positron emission tomography-computed tomography (PET-CT) showed accumulation in the upper mediastinal tumor (SUVmax = 5.18), the left 1^st^ and 4^th^ ribs (SUVmax = 5.48), and lumbar vertebrae L1 and L2 (SUVmax = 7.64), but no hot spot in the thyroid (Figure 1(b)). A bone biopsy of the lumbar vertebrae revealed atypical epithelial cells with a follicular structure and nuclear groove on HE staining, and immunostaining was positive for thyroglobulin and PAX-8 (Figure 2), indicating the presence of a bone metastasis of papillary thyroid carcinoma.

Cervical ultrasonography showed no obvious malignancy in the thyroid. Tumor markers and thyroid function tests were almost normal without thyroglobulin elevation (103 ng/mL).

We performed total thyroidectomy to administer radioiodine therapy and to reveal any primary tumor present in the thyroid. However, we found no papillary carcinoma in the gland.

The mediastinal tumor seemed to be firmly attached to the superior vena cava, and congestion was strong because of superior vena cava syndrome. Therefore, removing the superior mediastinal tumor or obtaining a biopsy was impossible. However, we suspected that the upper mediastinal tumor was the primary site because we found no other tumors. After surgery, we performed radioactive iodine (RAI) therapy as planned. A dosage of 100 mCi was administered to the patient. Although we observed uptake into the thyroid floor, we found no evidence of uptake into the mediastinum or bone. The thyroglobulin decreased to 26.1 ng/mL after RAI therapy. One year after surgery, there was no tumor growth and the superior vena cava syndrome was improving with the development of collateral circulation.

**Figure 3:**
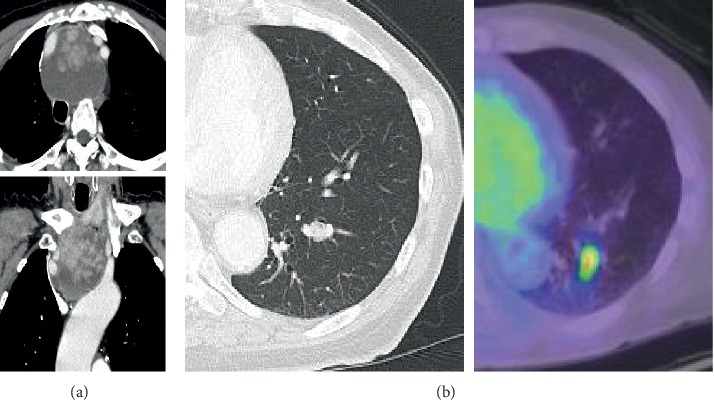
CT images. (a) 7.2 cm mass in the anterior mediastinum. (b) Pulmonary metastasis.

Case 2 is an 84-year-old man without remarkable medical history other than hyperlipidemia and prostatic hyperplasia. His chief complaint was cough, and an X-ray image showed an abnormal shadow on the upper mediastinum. A CT scan showed a 7.2 cm large mixed mass with a papillary solid component in the anterior mediastinum, and the trachea was compressed to the right and back. We could not confirm continuity with the thyroid; we found no lymph node metastases (Figure 3).

A PET-CT showed accumulation in the upper mediastinal tumor (SUVmax = 46.32) and pale nodules in the left lower lobe (SUVmax = 5.23; Figure 3). Neck ultrasonography revealed multiple nodules reminiscent of an adenomatous goiter in both lobes with no malignant appearance (the mediastinal tumors could not be visualized, and their continuity with the thyroid was unclear).

We performed endobronchial ultrasound-guided transbronchial needle aspiration (EBUS-TBNA) for diagnosis of the mediastinal tumor, but found no tumor cells, and the tumor's origin remained unknown.

In blood tests, the thyroid function was normal, but the thyroglobulin level rose to 2450 ng/mL. No other tumor markers were significant. We suspected that the mediastinal tumor originated from a thyroid cancer, and we decided to resect it.

The patient underwent total thyroidectomy, central neck dissection, and mediastinal tumor resection with sternal incision.

The mediastinal tumor adhered to the brachiocephalic artery and the right recurrent nerve on the right side, with the trachea and esophagus on the dorsal side and with the left recurrent laryngeal nerve on the left side. On the caudal side, a part of the thymus was resected together because the tumor also adhered to it.

**Figure 4:**
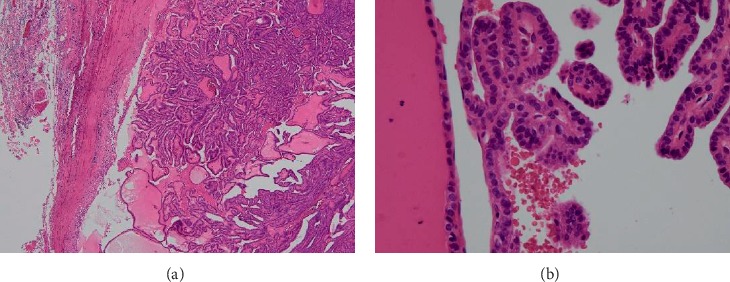
Pathology results for a tumor. Mediastinal tumor growing in papillary shape with a fibrotic capsule. Tumor cells have nuclear groove and inclusion body (HE staining). (a) ×20. (b) ×400.

Pathological findings included a fibrotic capsule and marked internal necrosis. The pathological report suggested a papillary carcinoma because of the preponderance of papillary growth and the presence of a papillary carcinoma nucleus (nuclear groove; Figure 4). We found no evidence of continuity between the mediastinal tumor and the thyroid. In addition, we noticed a 9 mm nodule in the isthmus of the thyroid and a nuclear finding of papillary carcinoma. We considered the nodule to represent a metastasis to the thyroid. We found no lymph node metastases. The thyroglobulin level decreased to 25.7 ng/mL after surgery. Administration of 131-iodine (100 mCi) resulted in strong accumulation in the thyroid bed and weak accumulation in lung metastasis. Therefore, we plan to follow-up the RAI therapy.

**Figure 5:**
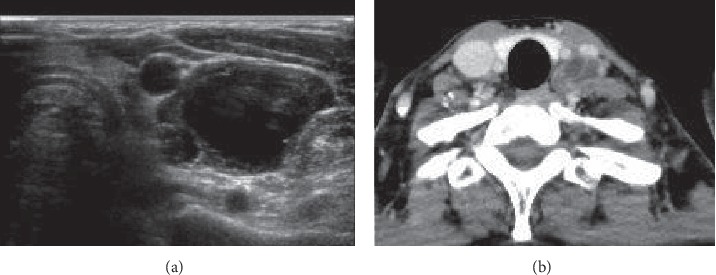
Left lateral cervical lymph node imaging. Neck ultrasonography (a) and CT scan (b) images showing cervical lymph node swelling.

Case 3 is a 56-year-old woman without remarkable medical history. Her chief complaint was a neck mass. We found swelling of the lateral cervical lymph nodes upon examination. Neck ultrasonography showed no thyroid malignancies. Contrast-enhanced CT showed a hepatic hemangioma only, without findings suggesting a primary site (Figure 5). Upper gastrointestinal endoscopy and transvaginal ultrasonography were both normal.

Blood tests showed no tumor marker or thyroid function abnormalities, the thyroglobulin level was 37.9 ng/mL, and anti-thyroglobulin antibody was negative. Fine needle aspiration cytology of the lymph node revealed an adenocarcinoma without nuclear findings of papillary carcinoma. However, the thyroglobulin value of the puncture fluid was high (>500 ng/mL).

**Figure 6:**
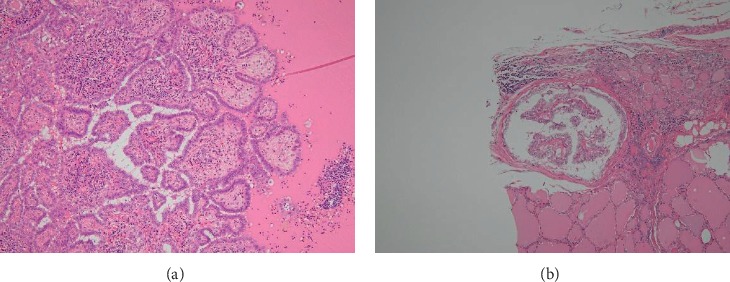
Microscopic pathology slides of left cervical lymph nodes. (a) Left cervical lymph nodes had nuclear findings of papillary thyroid carcinoma. (b) 1 mm papillary carcinoma tumor in the left lobe (HE staining ×100).

Since we suspected a cervical lymph node metastasis of thyroid cancer, we performed total thyroidectomy plus bilateral modified neck dissection to look for the primary tumor and for RAI therapy. A search through the excised thyroid with 4 mm slices revealed 1 mm of papillary carcinoma in the left lobe. We found metastases in 11 of 31 lymph nodes, with a maximum diameter of 23 mm. The metastatic lymph nodes had nuclear findings of papillary thyroid carcinoma (Figure 6).

The patient is considered to be at an ATA intermediate risk as she exhibited clinical N1 within all involved lymph nodes of <3 cm at their largest dimension [1]. She was recommended postoperative RAI, but she refused this procedure. TSH suppression therapy was performed, and TSH was suppressed under 0.1 *μ*U/ml. Thyroglobulin levels decreased to <1 ng/mL following surgery. The patient did not experience recurrences up to 4 postoperative years.

## 3. Discussion

Occult thyroid carcinoma is a general term indicating clinically different situations that can be classified into five types as described below:

Type 1 is sometimes found by autopsy or surgery for a benign lesion and is sometimes called latent cancer. Type 2 is a papillary thyroid microcarcinoma (PTMC) detected accidentally, mainly by ultrasonography. Type 3 is found with clinically apparent metastases of thyroid carcinoma, where the primary carcinoma is not detectable before surgery but is found in the final histological specimen. Type 4 originates from an ectopic thyroid gland. Type 5 has no carcinoma in the thyroid gland, only metastasis, also known as primary unknown cancer [2, 3].

The frequency of type 2 PTMC has increased because of improvements in diagnostic techniques such as ultrasonography [4], but the frequency of type 3 seems to have decreased. In addition, types 3, 4, and 5 are difficult to diagnose because no preoperative examination shows malignant thyroid findings. Therefore, these three types of occult cancer are clinically problematic. Of the three cases that we have encountered, patients with type 4 (cases 1 and 2) originated from ectopic thyroid gland tissue, and with type 3 (case 3) in an occult thyroid cancer. All of these are very rare cases.

Ultrasonography and fine needle aspiration cytology are useful and recommended for the diagnosis of thyroid cancers [1]. However, these diagnostic procedures are difficult in ectopic thyroid glands in the mediastinum because of anatomical complexity (presence of bones and lungs). We have encountered and reported two cases of patients with mediastinal tumors that were not thyroid cancers and were diagnosed after biopsy by bronchoscopy, immunohistological examination for PAX-8, thyroglobulin, and TTF-1 [5].

In case 1, we detected a papillary thyroid cancer from a bone metastasis biopsy. HE staining revealed an atypical epithelium and nuclear groove with follicular structure, and immunostaining was positive for thyroglobulin and PAX-8. Thus, we diagnosed the patient as having a metastasis of papillary thyroid cancer. The mediastinal tumor had displaced the superior vena cava, and the patient presented a superior vena cava syndrome. We thought that our patient presented a high bleeding risk because of the invasion of the superior vena cava and development of collateral circulation, and we decided not to resect the mediastinal tumor. We considered this ectopic thyroid as the primary tumor site because of the absence of another primary lesion (including in the thyroid gland).

In case 2, we performed EBUS-TBNA for a mediastinal tumor, but were not able to reach a diagnosis. We suspected a thyroid cancer because of the image findings and high blood thyroglobulin level. Thus, we performed both total thyroidectomy and mediastinal tumor resection by sternotomy. The mediastinal tumor pathological findings suggested a papillary thyroid cancer. A small papillary carcinoma was found in the thyroid gland, but the tumor volume was clearly smaller than the mediastinal tumor, and we think that the mediastinal tumor was the primary ectopic thyroid cancer.

Thus, we diagnosed the ectopic thyroid cancer by combining radiographic findings and immunostaining of specimens obtained by biopsy or surgery.

On the other hand, in case 3, we found no papillary thyroid cancer characteristics by cytology of the cervical lymph nodes. However, after measuring the thyroglobulin value of the puncture fluid, we found an adenocarcinoma derived from the thyroid gland. Uruno et al. reported that the measurements of thyroglobulin in the puncture fluid are useful for diagnosing cervical lymph node metastases in differentiated thyroid cancers [6]. Our results with case 3 support this idea. In addition, pathological examination of the thyroid gland after total thyroidectomy revealed a primary tumor in the thyroid gland, but its tumor size was so small that preoperative ultrasonography did not detect it.

Occult thyroid cancers in cervical lymph node metastases are intermediate to high risk, and RAI ablation should be considered [7]. If present in other metastases, RAI is recommended because distant metastases are considered an ATA high risk [1]. In cases where occult thyroid cancer is suspected, the metastasis site should be removed if possible for diagnostic and therapeutic purposes, and total thyroidectomy should be considered to allow for RAI, even if no thyroid gland findings are present.

Although few such cases have been reported, occult carcinoma exhibiting cervical cystic lymph node metastasis as the first manifestation has been reported. Verge et al. described seven papillary thyroid carcinomas that were discovered following a solitary lateral cervical cyst, and reported that ipsilateral modified neck dissection and total thyroidectomy followed by radioactive iodine therapy offered a favorable prognosis [8]. However, pathologic N1 with any metastatic lymph node >3 cm at its largest dimension is described as a high risk for ATA. When a tumor is discovered with cervical lymph node metastasis, the tumor size of lymph node metastasis is expected to be large. In high-risk cases, recurrence should be monitored, just as with regular PTC.

Occult papillary thyroid cancer is rare, and diagnosis is often impossible by ultrasonic examination or puncture aspiration cytology alone. Combining radiographic images, blood tests, immunohistological tests, and clinical signs is necessary. If occult thyroid cancer is suspected, total thyroidectomy should be considered even in the absence of malignant thyroid findings.
